# Tryptophan depletion in chronic fatigue syndrome, a pilot cross-over study

**DOI:** 10.1186/1756-0500-7-650

**Published:** 2014-09-16

**Authors:** Gerard KH The, Robbert J Verkes, Durk Fekkes, Gijs Bleijenberg, Jos WM van der Meer, Jan K Buitelaar

**Affiliations:** Department of General Internal Medicine, Nijmegen Expert Centre Chronic Fatigue, Radboud University Nijmegen Medical Centre, Reinier Postlaan 4, 6525 GC Nijmegen, The Netherlands; Department of Psychiatry, Donders Institute for Brain Cognition and Behaviour, Radboud University Nijmegen Medical Centre, Reinier Postlaan 10, 6525 GC Nijmegen, The Netherlands; Pompestichting for Forensic Psychiatry, Weg door Jonkerbos 55, 6532 CN Nijmegen, The Netherlands; Department of Psychiatry and Clinical Chemistry, Erasmus MC, dr. Molewaterplein 50, 3015 GE Rotterdam, The Netherlands; Department of Neuroscience, Donders Institute for Brain Cognition and Behaviour, Radboud University Nijmegen Medical Centre, Kapittelweg 29, 6525 GC Nijmegen, The Netherlands

**Keywords:** Serotonin, Tryptophan, Depletion, Fatigue

## Abstract

**Background:**

Chronic fatigue syndrome (CFS) is still an enigmatic disorder. CFS can be regarded as a complex disorder with tremendous impact on lives of CFS-patients. Full recovery without treatment is rare.

A somatic explanation for the fatigue is lacking. There is clinical and experimental evidence implicating enhanced serotonergic neurotransmission in CFS. Genetic studies and imaging studies support the hypothesis of upregulated serotonin system in CFS.

In line with the hypothesis of an increased serotonergic state in CFS, we performed a randomised clinical trial investigated the effect of 5-HT3 receptor antagonism in CFS. No benefit was found of the 5-HT3 receptor antagonist ondansetron compared to placebo.

To further investigate the involvement of serotonin in CFS we performed a placebo controlled cross over pilot study investigating the effect of Acute Tryptophan Depletion.

**Findings:**

Five female CFS-patients who met the US Center for Disease Control and Prevention criteria for CFS were recruited. There were two test days, one week apart. Each participant received placebo and ATD. To evaluate the efficacy of the ATD procedure tryptophan and the large neutral amino acids were measured. The outcome measures were fatigue severity, concentration and mood states.

ATD resulted in a significant plasma tryptophan to large neutral amino acid ratio reduction of 96%. There were no significant differences in fatigue-, depression and concentration between the placebo- and ATD condition.

**Conclusions:**

These first five CFS-patients did not respond to the ATD procedure. However, a much larger sample size is needed to draw final conclusions on the hypothesis of an increased serotonergic state in the pathophysiology of CFS.

**Trial registration:**

ISRCTN07518149

## Findings

### Introduction

Chronic fatigue syndrome (CFS) is characterized by severe disabling fatigue of at least six months duration that has led to considerable impairment in daily functioning. A somatic explanation for the fatigue is lacking [1].

In the pathophysiology of CFS, several lines of evidence support the hypothesis of an underlying dysfunction of the serotonergic (5-hydroxytryptamine, 5-HT) system. In 1987 Newsholme inaugurated a possible link between an increased serotonin metabolism and chronic fatigue. In healthy persons exercise results in an increased brain 5-HT metabolism in response to an increased delivery of plasma tryptophan, the precursor of serotonin [2].

In CFS patients, neuroendocrine challenge studies have suggested both an increased [3] and normal [4] 5-HT function. These discrepancies might be explained by differences in methodology, composition of the patient cohort and the use of different 5-HT agonists [5]. Two positron emission tomography studies have investigated the serotonin system in CFS [6, 7]. Both studies suggest an increased serotonin activity in CFS. A genetic study, which evaluated the 5-HT system in CFS, reported the 5-HT receptor subtype HTR2a -1438 A polymorphism to be associated with CFS. This variation in HTR2a may result in enhanced activity of the 5-HT system and contribute to the pathophysiology of CFS [8].

In line with the hypothesis of an increased serotonergic state in CSF, we investigated the effect of a 5-HT3 receptor antagonist in a small open label study in 5 CFS-patients and found positive effects on fatigue severity and on functional impairment [9]. In a subsequent randomized clinical trial in 67 patients with CFS, no benefit was found of the 5-HT3 receptor antagonist ondansetron compared to placebo [10]. An earlier placebo-controlled double-blind study with the serotonin reuptake inhibitor fluoxetine showed no beneficial effect on any characteristic of CFS, including depressive symptoms [11].

To further investigate the involvement of 5-HT in CFS, we performed a placebo controlled cross over pilot study investigating the effect of Acute Tryptophan Depletion (ATD).

ATD is a reliable and reversible challenge test for serotonin and has been widely used over the last decade to investigate the role of serotonin in a variety of psychiatric disorders [12]. We postulate that ATD would have a positive effect in CFS-related symptoms such as fatigue severity, concentration and mood.

## Methods

The study was approved by the medical ethical committee of the Radboud University Nijmegen Medical Centre. Written informed consent was obtained from the participants.

Five female outpatients (age mean 24.0, SD 3.5 year, range 19 to 29 year) who met the US Center for Disease Control and Prevention criteria for CFS, were recruited through the outpatient clinic of the Department of General Internal Medicine of the Radboud University Nijmegen Medical Centre. A structured Clinical interview for DSM-IV (SCID-I) was performed by the researcher, to exclude patients with current psychiatric co-morbidity. Exclusion criteria were: vegetarians, nursing women, pregnant women, use of psychotropic medication in the last month, previous or current engagement in CFS or psychiatric research. Patients were tested in the follicular phase.

We used ATD mixtures and placebo mixtures as described by Sobczak et al. 2002 [13]. There were two test days, one week apart. On each test day, patients were assessed after an overnight fast. 24 hours before the test days patients had a tryptophan low diet [12]. Each participant received placebo and ATD. Baseline assessments (T0) took place at 8.30 a.m and five hours after ingestion of the mixtures the second assessment (T1) took place. The hospital pharmacy prepared the mixtures and the order of treatment was balanced over two the test days. The participants, investigators and laboratory technicians were blinded to the treatment condition.

To evaluate the efficacy of the ATD procedure, at T0 and T1, total plasma Tryptophan and LNAAs (phenylalanine, tyrosine, isoleucine, leucine and valine) were measured simultaneously after completion of the study with high-performance liquid chromatography as described elsewhere [14].

### Outcome measures

Reliable and validated instruments were used. Current fatigue severity was measured by the Checklist Individual Strength, subscale fatigue severity [15]. The score on this eight-item scale ranges from eight (no fatigue) to 56 (maximally fatigued). Current concentration was measured by the subscale CIS-concentration. The subscale consists of 5 items; range five (no concentration problems) to 35 (severe concentration problems).

The Dutch validated, shortened version of the Profile of Mood States (POMS), was used to assess mood changes during ATD. The subscale depression was used and current mood was assessed by a 5-point Likert Scale. The subscale depression consists of 8 items and ranges from 8 (no depression symptoms) to 40 (high level of depression symptoms). The Dutch POMS is considered a reliable instrument (Cronbach's alpha subscale depression 0.87). Strong correlations with other mood state and measures of depression have been found and the validity of the Dutch POMS is considered good [16, 17]. Current fatigue, concentration and mood were assessed at baseline (T0) and 5 hours after ingestion of the mixture (T1).

The outcome measures will be presented by means and standard deviation. A GLM repeated measures analysis will be performed using subject factors; time and ATD/ placebo.

## Results

All patients drank the ATD- and placebo mixtures within 15 minutes. Besides a little discomfort during and just after drinking the mixtures, the patients could tolerate the mixtures quite well.

ATD resulted in a significant plasma tryptophan to large neutral amino acid ratio reduction of 96% (data not shown). There were no significant differences in fatigue-, depression and concentration between the placebo- and ATD condition. Also, at the individual level, none of the patients presented with clear clinical improvements in the depleted condition (Table 1).

**Table 1:** **Outcome measures, Mean (sd), p-value**

Outcome	Treatment	T0 mean (sd)	T1 mean (sd)	p-value
CIS-fatigue	Placebo	36.4 (11.9)	42.2 (7.2)	0.97 (F = 0.002, df = 1)
	ATD	40.0 (7.2)	46.0 (3.9)	
CIS-concentration	Placebo	20.0 (10.6)	23.2 (9.9)	0.26 (F = 1,751, df = 1)
	ATD	22.4 (11.4)	23.0 (11.8)	
POMS-depression	Placebo	8.6 (0.6)	9.6 (0.6)	0.37 (F = 1, df = 1)
	ATD	9.6 (2.5)	9.4 (2.6)	

## Discussion

Different lines of evidence support the hypothesis of an increased serotonergic state in CFS. In this pilot study we evaluated the effect of an overall serotonin depletion in a cross-over design in CFS-patients. Serotonin depletion was achieved by acute depletion of the serotonin precursor tryptophan.

There is some evidence that there is a threshold for tryptophan depletion that needs to be exceeded before behavioral effects occur [18]. Although ATD resulted in a significant (96%) reduction of the tryptophan/LNAA ratio, no improvements were found in CFS-related symptoms. Since ATD very probably resulted in a transient serotonin depletion for a few hours, one could hypothesize that a more prolonged 5-HT depletion would be necessary to achieve neurobiological 5-HT changes and changes in fatigue severity in a chronic condition like CFS. A prolonged overall increased serotonergic synaptic neurotransmission could lead to a chronic downregulation of 5-HT1a receptors and a reduced binding potential [7]. Only a short and transient serotonin depletion would probably not lead to structural changes at the level of serotonin receptors. Furthermore, the most consistent finding of ATD is a mood-lowering response in patients recovered from depression [19]. To our knowledge, studies have not yet been performed investigating ATD effects on chronic fatigue in CFS patients; it is possible that ATD has a smaller impact in these patients. On the other hand, ATD studies have reported fast and significant effects on mood patients recovered from depression, particularly in those taking serotonergic antidepressants [20] and in patients with social anxiety disorder [20, 21]. Assessment took place 5 hours after ingestion of the mixtures. Plasma tryptophan reaches its minimum level between 5 to 7 hours after ingestion of the ATD mixture. It can be understood that there is a delay from the time plasma tryptophan reaches the point of maximal depletion to the time of maximum serotonergic depletion in the brain. In this study it is not exactly known when maximum effects occur. The study was performed in a homogeneous group of medication-free female CFS-patients. To avoid confounding effects of current psychiatric conditions that can affect brain serotonergic function we recruited CFS patients without current axis-1 psychiatric comorbidity. There is a group of subjects who are vulnerable to the effects of ATD. Patients who had previous episodes of depression or people with a family history of depression are vulnerable [20]. In our study we did not screen in detail the patients history or the family history. However any undetected vulnerability for depression would have increased the chance for finding ATD would lead to lowering of mood. In fact, in the study we did not observe a mood lowering effect of ATD. A major limitation of this study is the small sample size. Although there was no change in any of the outcome measures and robust improvements at the individual level were not detected either, further research in larger sample sizes is needed to allow a more definite answer, regarding the effect of ATD in CFS. In conclusion, our earlier treatment study with a selective serotonin blocking agent as well as the present study with alimentary induction of significant ATD showed no positive results in any of the outcome measures in the treatment of CFS. Administering of ATD in a much larger sample size is needed to draw final conclusions on the hypothesis of an increased serotonergic state in the pathophysiology of CFS.

## Abbreviations

5-HT: 5-hydroxytryptamine
ATD: Acute tryptophan depletion
CFS: Chronic fatigue syndrome
SCID-1: Structured clinical interview for DSM-IV.
